# Overexpression of *MdATG8i* Enhances Drought Tolerance by Alleviating Oxidative Damage and Promoting Water Uptake in Transgenic Apple

**DOI:** 10.3390/ijms22115517

**Published:** 2021-05-24

**Authors:** Xin Jia, Xiaoqing Gong, Xumei Jia, Xianpeng Li, Yu Wang, Ping Wang, Liuqing Huo, Xun Sun, Runmin Che, Tiantian Li, Yangjun Zou, Fengwang Ma

**Affiliations:** 1 State Key Laboratory of Crop Stress Biology for Arid Areas, Shaanxi Key Laboratory of Apple, College of Horticulture, Northwest A&F University, Yangling 712100, China; jiaxin09041005@163.com (X.J.); gongxq0103@nwsuaf.edu.cn (X.G.); jxm66660520@163.com (X.J.); 1827589945@163.com (X.L.); wy199649@163.com (Y.W.); tingtlc007@nwsuaf.edu.cn (P.W.); liuqingsugar@163.com (L.H.); 15929802107@163.com (R.C.); ltt15691091668@163.com (T.L.); 2 Center of Pear Engineering Technology Research, State Key Laboratory of Crop Genetics and Germplasm Enhancement, College of Horticulture, Nanjing Agricultural University, Nanjing 210095, China; sunxun1991@njau.edu.cn

**Keywords:** apple, autophagy, *MdATG8i*, drought tolerance, oxidative damage, flavonoid

## Abstract

Water deficit adversely affects apple (*Malus domestica*) productivity on the Loess Plateau. Autophagy plays a key role in plant responses to unfavorable environmental conditions. Previously, we demonstrated that a core apple autophagy-related protein, *MdATG8i*, was responsive to various stresses at the transcript level. Here, we investigated the function of this gene in the response of apple to severe drought and found that its overexpression (OE) significantly enhanced drought tolerance. Under drought conditions, *MdATG8i*OE apple plants exhibited less drought-related damage and maintained higher photosynthetic capacities compared with the wild type (WT). The accumulation of ROS (reactive oxygen species) was lower in OE plants under drought stress and was accompanied by higher activities of antioxidant enzymes. Besides, OE plants accumulated lower amounts of insoluble or oxidized proteins but greater amounts of amino acids and flavonoid under severe drought stress, probably due to their enhanced autophagic activities. Particularly, *MdATG8i*OE plants showed higher root hydraulic conductivity than WT plants did under drought conditions, indicating the enhanced ability of water uptake. In summary, the overexpression of *MdATG8i* alleviated oxidative damage, modulated amino acid metabolism and flavonoid synthesis, and improved root water uptake, ultimately contributing to enhanced drought tolerance in apple.

## 1. Introduction

Apple (*Malus domestica* Borkh.) is one of the most important fruit crops worldwide and is widely cultivated in temperate regions. Currently, the Loess Plateau is a main apple production region in China, owing to its high altitude, sufficient light, and wide temperature variation. However, drought stress is becoming severe and seriously restricts apple production in the Loess Plateau region due to light and uneven annual rainfall. Sustainable agricultural development urgently requires strategies and technologies for the improvement of apple drought tolerance.

Drought stress has adverse impacts on various aspects of plant physiology, including photosynthesis, respiration, nutrient absorption, energy metabolism, and enzyme activity: it eventually reduces the growth, development, and productivity of many crops [1,2]. Drought stress typically causes osmotic stress, leading to a loss of cell turgor that greatly inhibits cell expansion and division, ultimately slowing plant growth [3]. Water deficits can also induce oxidative stress in plants because of the overaccumulation of ROS that seriously damages various cellular components, resulting in cell death and metabolic disturbance [4].

Over time, plants have developed various strategies to deal with water scarcity, involving molecular, physiological, and biochemical modifications [5]. Under drought conditions, plants maintain tissue water status primarily by closing stomata to reduce water loss and developing their root systems to increase water uptake [6,7]. For example, silicon application improved drought tolerance by increasing root water uptake in sorghum plants [8]. Plants can also improve osmotic adjustment to promote the maintenance of cell turgor: this can be achieved by the accumulation of osmolytes, including soluble sugars, free amino acids, organic acids, and glycine-betaine [9,10]. In addition, excess ROS generated during drought stress can be scavenged by enzymatic and non-enzymatic mechanisms. The activities of antioxidant enzymes such as superoxide dismutase (SOD), peroxidase (POD), and catalase (CAT) can be activated by drought. Non-enzymatic antioxidants such as glutathione, ascorbate, and flavonoid also accumulate in response to drought stress [11,12]. A recent report demonstrated that Heat Shock Factors (HSF) isolated from *Malus domestica*, designated MdHSFA8a, positively regulated apple drought tolerance by promoting flavonoid synthesis [13]. All these protective modifications can contribute to alleviating the adverse influence of drought stress.

As an evolutionarily conserved self-degradation mechanism in eukaryotes, autophagy delivers damaged proteins and cellular organelles to the vacuoles or lysosome for degradation, yielding amino acids, fatty acids, and sugars for reuse [14,15]. Autophagy controls protein quality under stress conditions, and increasing evidence suggests that it is crucial for plant responses to abiotic and biotic stresses, including drought [16,17,18,19]. In plants, autophagy helps to remove cellular components damaged by environmental stress [20,21]. Various *Arabidopsis* autophagy-deficient plants exposed to drought or salt stress exhibit increased stress sensitivity and contain greater levels of oxidized proteins compared with WT plants [22,23]. In tomato, the heat-shock transcription factor A1a (*HsfA1a*) enhances drought tolerance by binding directly to the promoters of two autophagy-related genes (*SlATG10* and *SlATG18f*) and inducing autophagy, which improves plant survival by degrading insoluble protein aggregates under drought stress [18]. In addition, a recent study in *Arabidopsis* found that a plant-specific DUF641 family protein, named Constitutively Stressed 1 (AtCOST1), balanced plant growth and drought tolerance by regulating autophagy [24].

In yeast, the ATG8 protein, a crucial component of the autophagic process, can bind to phosphatidylethanolamine (PE) with the help of ATG4, ATG7, and ATG3 proteins [25]. The ATG8–PE conjugate, anchoring on the membranes of autophagosomes until autophagic bodies degrade in vacuoles, participates in the extension and formation of the autophagosome and is considered to be a marker of the autophagy process [26]. ATG8–PE also plays a key role in selective autophagy [27]. It can interact with cargo receptors to recognize specific proteins for degradation [27]. *Arabidopsis* has nine ATG8 isoforms (a–i), which are highly similar to yeast ATG8 [26,28]. Previously, we isolated the apple autophagy-related gene *MdATG8i* and found that this gene was responsive to various stresses and leaf senescence [29]. Here, we used *MdATG8i*OE apple plants to investigate the biological function of *MdATG8i* under drought stress. Overexpression of *MdATG8i* improved apple drought resistance by reducing ROS accumulation, promoting degradation of oxidized proteins, increasing accumulation of flavonoid, and maintaining root hydraulic conductivity. These changes were probably due to enhanced autophagic activities. Thus, the present study provides valuable information on the association between autophagy and apple drought tolerance.

## 2. Results

### 2.1. Overexpression of MdATG8i Increased Apple Plants’ Tolerance to Extreme Drought Stress

**Figure 1:**
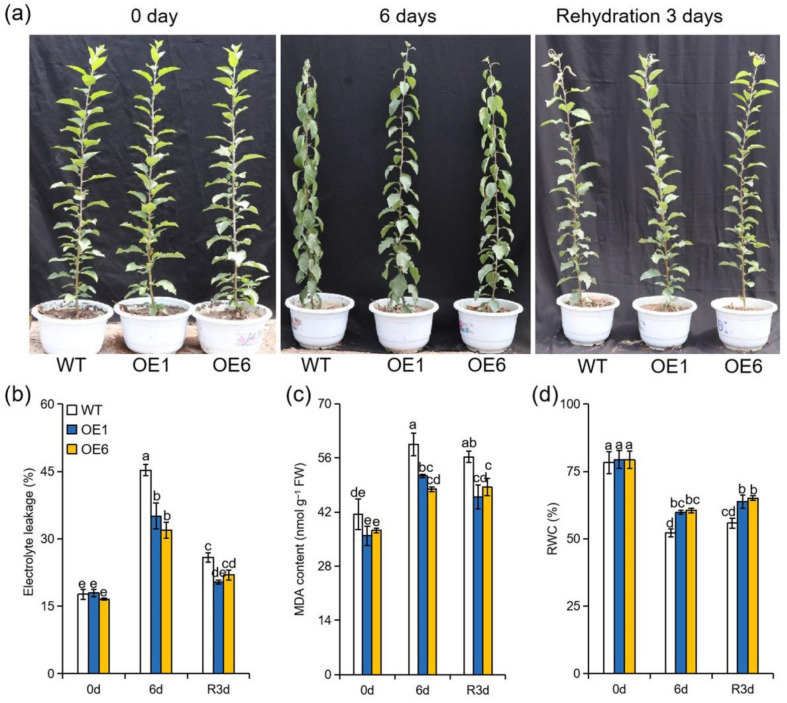
*MdATG8i* positively regulates drought tolerance. (**a**) Performance, (**b**) relative electrolyte leakage, (**c**) MDA content, and (**d**) RWC in the leaves of *MdATG8i*OE plants and WT. The data are presented as means ± SD of three replicates. Values with different letters are significantly different (*p* < 0.05, Tukey’s multiple range test).

In GL-3 plants (*Malus domestica* Borkh. cv. ‘Roya Gala’), the expression of *MdATG8i* could be induced by extreme drought (Figure S1). To further investigate the biological function of *MdATG8i* in response to drought, we used two previously obtained transgenic lines of *MdATG8i*OE apple [30]. Prior to the water-deficit treatment, there were no phenotypic differences between WT and *MdATG8i*OE lines. After six days of withholding water, leaves of WT plants showed more obvious symptoms of dehydration than leaves of OE plants. As shown in Figure 1a, the OE lines exhibited much less wilting. Most leaves of the OE plants recovered after rehydration, with the exception of their top two or three young leaves. By contrast, approximately six of the WT top young leaves remained dried after rehydration (Figure 1a). Multiple physiological parameters, such as RWC, electrolyte leakage, and MDA content, can be used to evaluate plant abiotic stress tolerance. The values of these parameters were similar in all genotypes under well-watered conditions. However, electrolyte leakage and MDA content were lower in OE lines than these in WT under drought and during rehydration (Figure 1b,c). Under drought stress, there was a greater reduction in RWC in WT than that in the *MdATG8i*OE lines. After re-watering, the RWC of WT plants returned to only 71.2% of the control value, whereas the OE lines reached 80.4–82% of the control (Figure 1d). All of these results demonstrated that overexpression of *MdATG8i* conferred enhanced tolerance to extreme drought in apple.

### 2.2. MdATG8i Overexpression Apple Plants Maintained Higher Photosynthetic Capacity under Drought Stress

**Figure 2:**
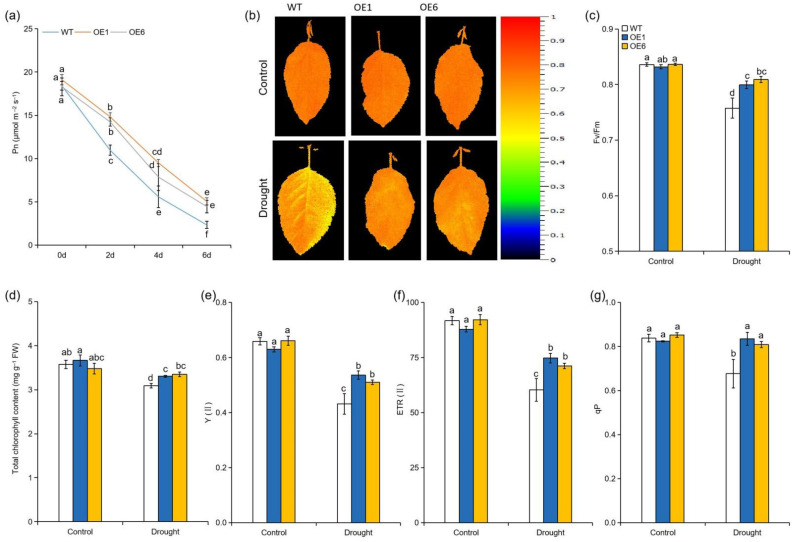
Effect of *MdATG8i* overexpression in apple on the photosynthetic and chlorophyll fluorescence parameters under drought stress. (**a**) Photosynthetic rate (Pn), (**b**) chlorophyll fluorescence images, (**c**) the maximum quantum yield of PSII (Fv/Fm), (**d**) total chlorophyll content, (**e**) the effective quantum yield of PSII [Y(II)], (**f**) the electron transport rate of PSII [ETR (II)], and (**g**) photochemical quenching (qP) of WT and *MdATG8i*OE plants with or without drought stress. The data are presented as means ± SD of three replicates. Values with different letters are significantly different (*p* < 0.05, Tukey’s multiple range test).

Drought stress can directly reduce photosynthetic efficiency, eventually inhibiting plant growth [31]. To explore the photosynthetic performance of WT plants and OE lines under drought conditions, we measured net photosynthesis rate, chlorophyll content, and chlorophyll fluorescence parameters. Under normal conditions, no significant differences in these parameters among the genotypes were observed between WT and *MdATG8i*OE lines (Figure 2a–g). During drought period, the levels of Pn were gradually reduced in all genotypes, but the reduction was greater in WT plants than in the *MdATG8i*OE plants (Figure 2a). After 6 d of water deprivation, chlorophyll contents were decreased in all genotypes, but they were still higher in the *MdATG8i*OE lines than in WT (Figure 2d). Furthermore, drought stress significantly damaged chlorophyll fluorescence in all genotypes. However, WT plants showed more significant declines in *Fv/Fm*, Y(II), ETR(II), and qP under drought conditions when compared with *MdATG8i*OE plants (Figure 2b,c,e–g). All of these results suggested that *MdATG8i*OE plants maintained better photosynthetic capacity under drought conditions.

### 2.3. Overexpression of MdATG8i in Apple Stimulated ROS Scavenging under Drought Stress

**Figure 3:**
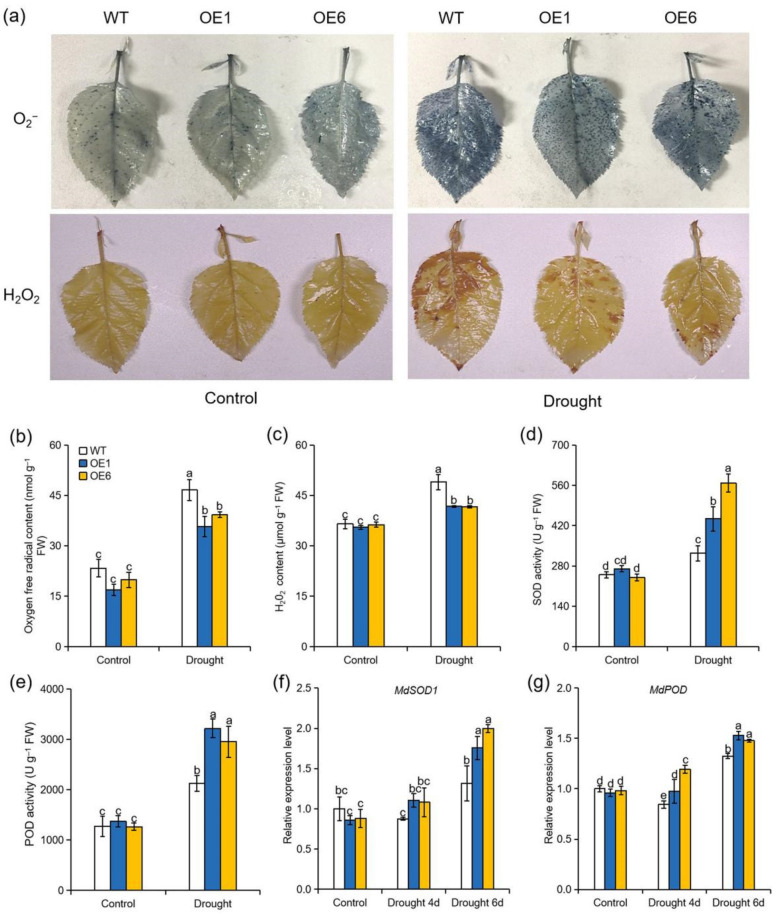
Effect of *MdATG8i* overexpression in apple on ROS scavenging under drought conditions. (**a**) Detection of O_2_^−^ and H_2_O_2_ by NBT and DAB staining, (**b**) O_2_^−^ concentrations, (**c**) H_2_O_2_ concentrations, (**d**) SOD activity, and (**e**) POD activity in the leaves of WT and *MdATG8i*OE plants. Changes in the expression levels of (**f**) *MdSOD1* and (**g**) *MdPOD* after drought stress. The data are presented as means ± SD of three replicates. Values with different letters are significantly different (*p* < 0.05, Tukey’s multiple range test).

High levels of toxic ROS accumulation under drought stress can result in oxidative stress that ultimately damages cellular components. At the end of the drought treatment, leaves were harvested to be stained using NBT and DAB to examine O_2_^−^ and H_2_O_2_, respectively. The extents of NBT and DAB staining were similar in WT and OE plants under normal conditions. However, all genotypes were stained extensively under drought stress, but denser staining was observed in the WT plants when compared with OE lines (Figure 3a). Consistently, quantitative measurements of O_2_^−^ and H_2_O_2_ contents suggested that the *MdATG8i*OE lines accumulated fewer ROS compared with WT under severe drought (Figure 3b,c). Besides, the activities of SOD and POD were significantly elevated under drought stress in all genotypes, but greater increases were observed in the OE plants than that in WT (Figure 3d,e). The expression levels of genes encoding SOD1 and POD were elevated more in the OE lines compared with the WT plants under drought stress (Figure 3f,g). These results indicated that overexpression of *MdATG8i* positively regulated the antioxidant capacity in apple under drought conditions.

### 2.4. Overexpression of MdATG8i in Apple Reduced the Accumulation of Insoluble or Oxidized Proteins under Drought Stress

**Figure 4:**
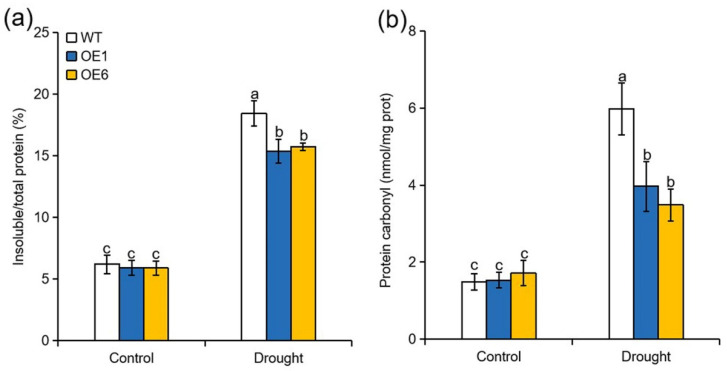
Effect of *MdATG8i* overexpression in apple on the accumulation of insoluble and oxidized proteins under drought conditions. (**a**) Percentage of insoluble proteins relative to total proteins and (**b**) levels of oxidized proteins in WT and *MdATG8i*OE plants. The data are presented as means ± SD of three replicates. Values with different letters are significantly different (*p* < 0.05, Tukey’s multiple range test).

Autophagy functions on degrading damaged proteins and organelles, which are generated under unfavourable environment [20,32,33]. To analyze whether overexpression of *MdATG8i* influence the accumulation of damaged proteins, we determined the amount of insoluble or oxidized proteins in the WT and *MdATG8i*OE plants when exposed to drought. As shown in Figure 4a, no significant difference in the amount of insoluble protein as a percentage of the total was observed between WT and transgenic plants under normal conditions. However, the amount of insoluble proteins were approximately 20% and 17% higher in WT than in OE1 and OE6 plants, respectively, at the end of the drought treatment (Figure 4a). Furthermore, carbonyl protein contents were elevated in all genotypes under drought treatment, but the accumulation of oxidized protein was partially alleviated by *MdATG8i* overexpression (Figure 4b). These results suggested that overexpression of *MdATG8i* in apple promoted the degradation of oxidized or insoluble proteins under drought stress.

### 2.5. Overexpression of MdATG8i Modulated Amino Acid Metabolism under Drought Stress

**Figure 5:**
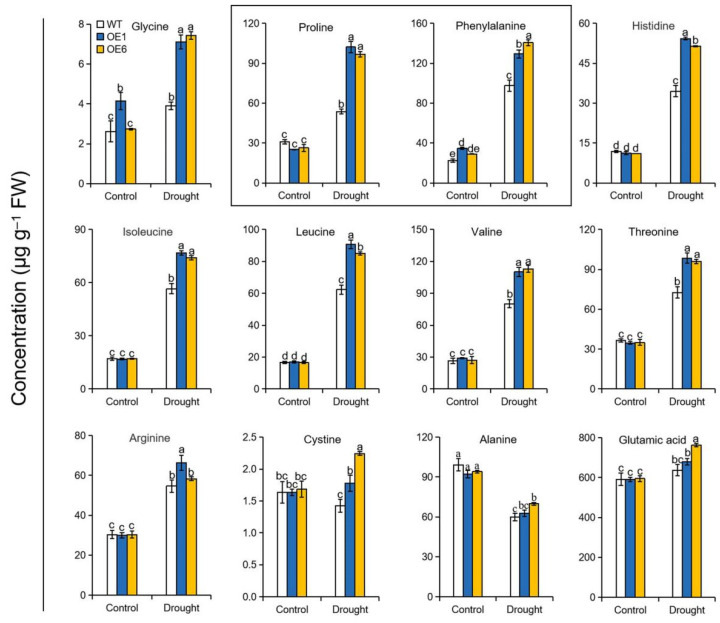
Accumulation of amino acids in the leaves of WT and *MdATG8i*OE plants under drought conditions. FW, fresh weight. The data are presented as means ± SD of three replicates. Values with different letters are significantly different (*p* < 0.05, Tukey’s multiple range test).

Based on the differences in protein degradation observed above, we next measured the amino acid metabolism of WT and OE plants subjected to drought. As shown in Figure 5, the levels of most measured amino acids were raised in response to drought stress among all genotypes. Furthermore, levels of these responsive amino acids were elevated to a greater extent in the OE lines than in WT plants under drought conditions. For example, the proline concentrations were approximately 2-fold higher in OE lines than in WT plants. In addition, the concentrations of glycine, histidine, and phenylalanine were approximately 1.9, 1.5, and 1.4 times higher in the *MdATG8i*OE plants than in WT when exposed to drought. These results demonstrated that overexpression of *MdATG8i* in apple improved amino acid metabolism in response to drought.

### 2.6. Overexpression of MdATG8i Promoted the Synthesis of Flavonoid under Drought Stress

**Figure 6:**
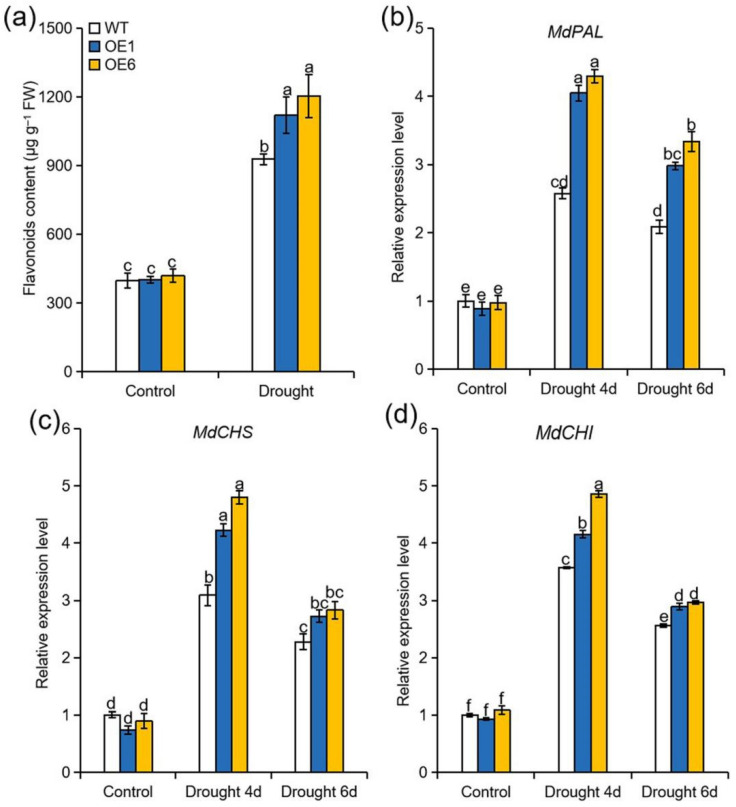
Effect of *MdATG8i* overexpression in apple on the accumulation of flavonoids under drought stress. (**a**) Flavonoid content in leaves of WT and *MdATG8i*OE plants. Expression levels of (**b**) *MdPAL*, (**c**) *MdCHS*, and (**d**) *MdCHI* in the leaves of WT and *MdATG8i*OE plants. The data are presented as means ± SD of three replicates. Values with different letters are significantly different (*p* < 0.05, Tukey’s multiple range test).

Phenylalanine is the precursor for flavonoid biosynthesis in plants [34]. Due to significant differences in phenylalanine content between OE and WT plants under drought stress, we next measured the levels of flavonoid. Consistent with the trend in phenylalanine content, higher levels of flavonoid were observed in *MdATG8i*OE lines than in WT after drought treatment (Figure 6a). *Phenylalanine ammonia lyase* (*PAL*), *chalcone synthase* (*CHS*), and *chalcone isomerase* (*CHI*) are common early genes in the pathway of flavonoid synthesis, and we therefore examined their transcript levels [35]. Transcript levels of *MdPAL*, *MdCHS*, and *MdCHI* were upregulated more in the *MdATG8i*OE lines than these in the WT plants when exposed to drought (Figure 6b–d). Therefore, overexpression of *MdATG8i* in apple might contribute to enhancing the synthesis of flavonoid under drought stress.

### 2.7. Overexpression of MdATG8i Improved Root Vitality and Hydraulic Conductivity of Apple Plants under Drought Stress

**Figure 7:**
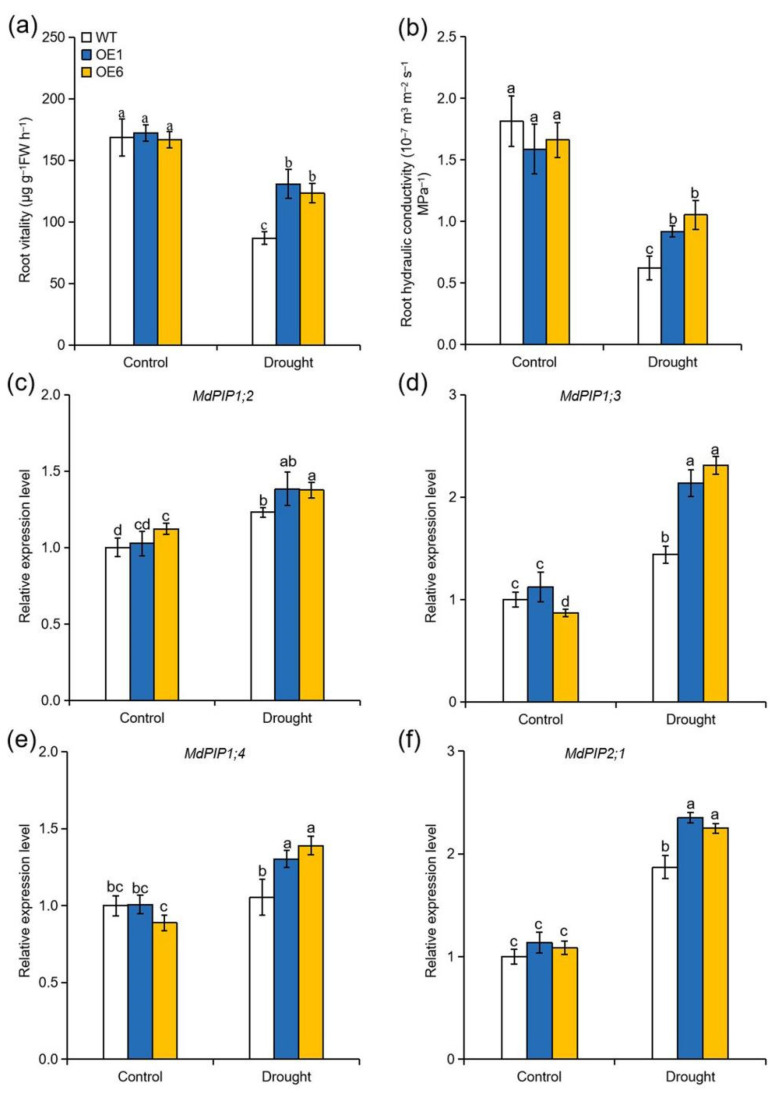
Effect of *MdATG8i* overexpression in apple on root water uptake under drought conditions. (**a**) Root vitality and (**b**) root hydraulic conductivity of the WT and *MdATG8i*OE plants after 4 d of drought treatment. Change in the expression levels of (**c**) *MdPIP1;2*, (**d**) *MdPIP1;3*, (**e**) *MdPIP1;4*, and (**f**) *MdPIP2;1* in the roots of WT and transgenic plants after 4 d of drought treatment. The data are presented as means ± SD of three replicates. Values with different letters are significantly different (*p* < 0.05, Tukey’s multiple range test).

Roots are the key to solving many problems associated with drought stress, as they play a crucial role in the modulation of water status of plant. Root vitality is a widely used physiological indicator that represents the overall activity of the root system and root hydraulic conductivity reflects the capacity for water uptake and transport by roots [36]. Root hydraulic conductivity and root vitality were therefore measured to determine whether *MdATG8i* influences the modulation of root water uptake under drought stress. Under normal conditions, there were no significant differences in root hydraulic conductivity and root vitality among the genotypes. However, both parameters declined sharply under drought treatment in all genotypes. Nonetheless, the root hydraulic conductivity and root vitality of WT plants were significantly lower than those of the OE lines after 4 d of drought treatment (Figure 7a,b). Plasma membrane intrinsic proteins (PIPs) are members of aquaporin, which function on regulating water transport across membranes [37]. Consistent with the higher root water uptake in the OE lines in response to drought, the expression levels of *MdPIP1;2*, *MdPIP1;3*, *MdPIP1;4*, and *MdPIP2;1* were higher in the OE lines than these in the WT plants under drought conditions (Figure 7c–f). These results demonstrated that *MdATG8i*OE plants maintained better root water absorption capacity under drought conditions.

### 2.8. Overexpression of MdATG8i in Apple Enhanced the Transcript of other Autophagy-Related (ATG) Genes and the Accumulation of Autophagic Structures under Drought Stress

**Figure 8:**
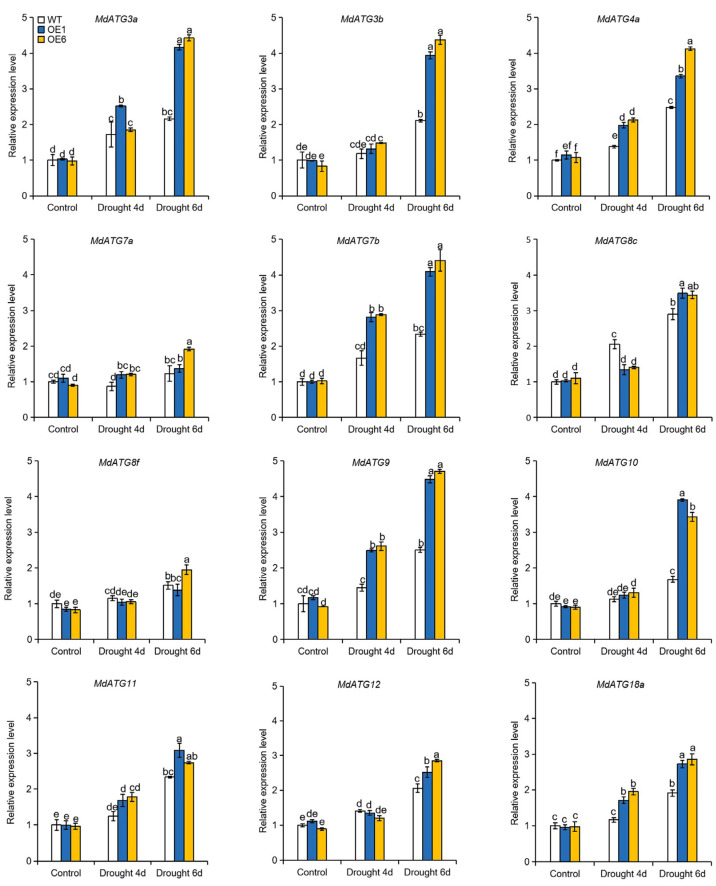
Change in the expression levels of several core apple autophagy-related genes (*MdATGs*) in the leaves of the WT and transgenic plants following drought treatment. The data are presented as means ± SD of three replicates. Values with different letters are significantly different (*p* < 0.05, Tukey’s multiple range test).

**Figure 9:**
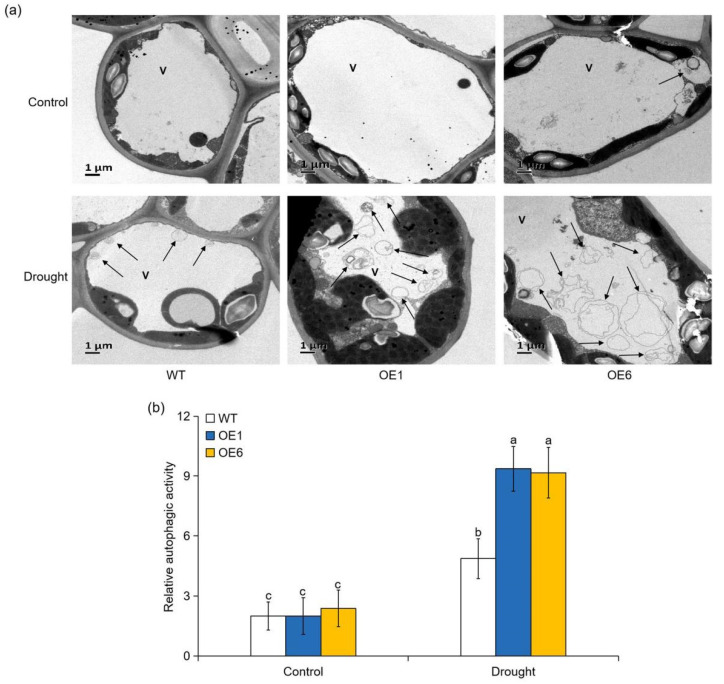
Effect of *MdATG8i* on the accumulation of autophagic structures in apple under drought conditions. (**a**) Representative images of autophagic structures in leaf mesophyll cells of WT and transgenic plants observed by TEM. V, vacuole. Autophagic structures are indicated by arrows. Scale bars: 1 μm. (**b**) Relative autophagic activity quantified from the leaves of WT and *MdATG8i*OE plants as shown in (**a**). The data are presented as means ± SD of ten replicates. Values with different letters are significantly different (*p* < 0.05, Tukey’s multiple range test).

To investigate autophagic activity under drought stress, we measured the transcript levels of 12 core autophagy genes. As shown in Figure 8, the expression levels of these *ATG* genes were similar in OE lines and WT plants under normal growth conditions. Drought induced the expression of almost all the *ATG* genes in all genotypes, but their expression levels were significantly higher in the OE plants than in the WT plants (Figure 8). To further confirm the differences in drought-induced autophagy among the genotypes, transmission electron microscopy (TEM) was used to detect autophagic activity. Under normal conditions, only a few autophagic structures were observed in all genotypes (Figure 9a,b). However, the number of autophagic structures increased by nearly 2.5-fold in the WT plants and by 3.9- to 4.6-fold in the OE1 and OE6 lines after 6 d of drought treatment (Figure 9a,b). These results indicated that overexpression of *MdATG8i* enhanced the occurrence of autophagy in apple when subjected to drought stress.

## 3. Discussion

Autophagy is a conserved eukaryotic process in which unwanted or damaged cellular components are transferred to vacuoles to be recycled; it has been shown to play critical roles in improving plant tolerance to various abiotic and biotic stresses [14,17,38]. We previously isolated the *ATG8* gene family member *MdATG8i* from *M. domestica* and found that its expression was responsive to oxidative stress, starvation, and leaf senescence [29]. In this study, we further explored its function in the regulation of apple drought tolerance. *MdATG8i* overexpression reduced drought damage to apple plants, as demonstrated by higher RWC levels and chlorophyll concentrations, lower REL and MDA levels, and improved photosynthetic capacity (Figure 1). *MdATG8i* appeared to enhance apple drought tolerance by limiting oxidative damage via increasing accumulation of flavonoid and activities of antioxidant enzymes, as well as by maintaining root hydraulic conductivity, all of which might be attributed to increased autophagic activity resulting from *MdATG8i* overexpression.

As key physiological processes, photosynthesis and photosynthetic electron transport can be affected by drought stress because of stomatal closure and the damage to the photosynthetic apparatus [39]. Toxic ROS that are generated under drought stress can inhibit ATP synthesis, Rubisco activation, and PSII repair, ultimately reducing photosynthetic performance [40]. Protection of the photosynthetic system is of primary importance in improving the ability of plants to withstand drought stress. Here, the improved photosynthetic capacities in the OE plants might be the results of reduced oxidative damage under drought stress (Figure 2).

Drought stress can induce the overproduction of ROS such as O_2_^−^ and H_2_O_2_. Excess ROS accumulation leads to oxidative stress that can damage cellular components such as lipids, proteins, DNA, and organelles, ultimately resulting in cell death [4]. Plants have evolved multifaceted ROS-scavenging mechanisms—including both enzymatic and nonenzymatic components—to eliminate toxic ROS [11,12]. SOD converts O_2_^−^ to H_2_O_2_, which is then scavenged by POD [41]. Jia et al. demonstrated that overexpression of *Malus domestica* NAC transcription factors 1 (*MdNAC1*) enhanced apple drought tolerance by promoting activities of SOD and POD [42]. Here, we found that *MdATG8i*OE apple plants had higher transcript levels of SOD and POD than WT plants under drought stress (Figure 3f,g). Accordingly, the activities of SOD and POD were also significantly higher in OE lines than in WT plants under drought (Figure 3d,e). Consistent with better ROS scavenging ability, the OE lines accumulated fewer ROS and less MDA than WT plants under drought stress (Figure 3a–c). The improved antioxidant system of the OE plants helped to alleviate oxidative damage and protect the photosynthetic apparatus, eventually resulting in enhanced drought tolerance. Our results were in agreement with previous reports in which plants that overexpressed autophagy genes exhibited greater tolerance to various stresses through enhanced ROS scavenging [30,33]. Improved ROS scavenging capacity in response to multiple stresses may be considered a common mechanism by which autophagy contributes to increased stress tolerance.

Drought can also induce the aggregation of misfolded and oxidized proteins that are toxic to plant cells, and autophagy enables the degradation of damaged proteins and organelles under various stress conditions [32,43]. In *Arabidopsis,* RNAi-*AtATG18a* plants have reduced tolerance to methyl viologen (MV)-induced oxidative stress because they accumulate higher amounts of oxidized proteins [20]. A similar phenotype has been observed in the rice *Osatg10b* mutant [21]. In addition, *Arabidopsis atg5* and *atg7* mutants are more sensitive to heat stress owing to the over-accumulation of insoluble proteins [44]. In tomato, *HsfA1a* induces drought tolerance by binding to the promoters of *ATG10* and *ATG18f* to activate autophagy, resulting in the degradation of ubiquitinated protein aggregates [18]. We also found that *MdATG8i* overexpression consistently promoted the degradation of insoluble and oxidized proteins in apple when subjected to drought (Figure 4). The enhanced occurrence of autophagy in OE plants was probably responsible for the reduced amounts of insoluble and oxidized proteins, thereby minimizing oxidative damage under drought stress.

In addition, osmotic stress can be induced when plants are subjected to drought stress, leading to a loss of cell turgor. Plants have evolved multiple strategies to mitigate the adverse effects of drought stress, and one such strategy involves amino acid metabolism [10]. The accumulation of amino acids that can act as compatible solutes to maintain osmotic equilibrium under drought has been observed in tomato, cotton, and maize [45,46,47]. Recent studies have suggested that autophagy helps to regulate the metabolism of free amino acids [48,49]. Here, the enhanced accumulation of amino acids in OE lines may have resulted from greater degradation of damaged proteins owing to greater autophagic activity under drought conditions (Figure 5). Furthermore, the levels of specific amino acids such as proline, glycine, and phenylalanine significantly differed between OE and WT plants during drought. Proline is reported to act as both an osmolyte and an ROS scavenger, alleviating damage caused by various stresses [50]. Overexpression of *Betula platyphylla* homeodomain-leucine zipper I class homeobox gene (*BpHOX2*) improved birch plants osmotic tolerance partly by promoting the biosynthesis of proline [51]. Here, in addition to its function in osmotic adjustment, proline may also have acted as a hydroxy radical scavenger to protect the photosynthetic apparatus under drought in OE lines. Therefore, the promotion of free amino acid content by *MdATG8i*, especially that of proline, improved the ability of apple plants to perform osmotic adjustment and ROS scavenging under drought stress, ultimately leading to improved drought tolerance.

Besides, phenylalanine acts as a key precursor for flavonoid biosynthesis [34]. Consistent with the observed changes in phenylalanine content, we also detected higher contents of total flavonoid in OE plants under drought (Figure 6). As typical antioxidants, flavonoid play a positive role in plant–environment interactions [52]. Because of their great capacity to remove ROS, the accumulation of flavonoid is activated by various environmental stresses [53]. It has been reported that *MdHSFA8a* overexpression enhances drought tolerance in apple mainly by promoting the biosynthesis of flavonoid [13]. Accumulated flavonoid in OE plants may have acted as antioxidants to scavenge ROS, thereby reducing oxidative damage [54]. The increased accumulation of flavonoid in the *MdATG8i*OE plants might contribute to the reduced oxidative damage caused by drought, ultimately being partly responsible for the enhanced drought tolerance. However, a definitive relationship between autophagy and increased contents of phenylalanine and flavonoid requires further exploration.

Roots are usually considered as the key to solve issues caused by soil water deficit [55,56]. Plants take up water and nutrients primarily through their roots, and these materials are transferred to shoots to participate in various processes. The capacity of the roots to transfer water from the surrounding soil can be assessed by measuring the root hydraulic conductivity [8]. Previous researches have shown that root hydraulic conductivity is correlated with drought tolerance. For example, MdMYB88 and MdMYB124 confer drought tolerance in apple by positively regulating root hydraulic conductivity [55]. Liu et al. demonstrated that ectopic expression of *OsPIP1;3* enhanced drought tolerance in transgenic tobacco by promoting root water uptake [57]. Overexpression of *SlPIP2;1*, *SlPIP2;7*, and *SlPIP2;5* conferred drought tolerance in tomato by elevating root hydraulic conductivity [58]. Here, compared with WT plants, higher root hydraulic conductivity of OE plants under drought stress indicated the improved ability of root water uptake, partly contributing to the maintenance of plant water balance (Figure 7).

## 4. Materials and Methods

### 4.1. Plant Materials and Treatments

Tissue-cultured plants of *M. domestica* ‘GL-3′ (‘Royal Gala’) were cultivated as described previously, and the expression levels of *MdATG8i* were increased by 5.4- and 10.1-fold in the OE1 and OE6 lines, respectively [30]. After 4 weeks on rooting medium, GL-3 and transgenic apple plants were moved to plastic bowls that contained a mixture of soil/perlite/roseite (3:1:1, *v*:*v*:*v*). After 4 weeks of acclimation in a growth chamber, apple plants were transferred to large pots (30 × 18 cm) filled with equal weight mixtures of soil/sand/organic matter (*v*:*v*:*v*, 5:1:1) and grown in a glasshouse that shared environmental conditions with the field at Northwest A&F University Yangling (34°20′ N, 108°24′ E), Shaanxi Province, China.

After 90 d of cultivation under well-watered conditions, similar sized plants from each line were divided into a normal water supply group and a drought treatment group. One day before the drought treatment, all plants were fully irrigated. Drought treatment was induced by withholding water for 6 days and re-watering followed this drought treatment. To evaluate the drought situation, the soil relative water content (SRWC) was monitored at 18:00 pm on day 0, 2, 4, 5, 6 of treatment using a weighting method (Figure S2). The SRWC was calculated as (Fresh pot weight–Dry weight)/(Maximum water content–Dry weight of soil) [59]. The SRWC of the normal water supply group were maintained at 75–85%. On days 0, 4, and 6, leaves 9–12 from the stem base were harvested from ten plants per treatment. The harvested leaves were dived into three replicates and stored in −80 °C.

### 4.2. RNA Extraction and qRT-PCR Analysis

Total RNA was isolated using a Wolact Plant RNA Isolation Kit (Wolact, Hong Kong, China). Quantitative real-time PCR (qRT-PCR) was performed with a LightCycler 96 quantitative instrument (Roche, Basel, Switzerland) and SYBR^®^ Premix Ex Taq^TM^ II (Takara). *M. domestica* malate dehydrogenase (*MdMDH*) was used as an internal control to standardize the cDNA samples for different genes. The primer sequences used in the expression analysis are listed in Table S1. Gene expression levels were calculated by the 2^–ΔΔCT^ method.

### 4.3. Analysis of Physiological Traits and Measurement of Root Hydraulic Conductivity

Relative electrolyte leakage (REL) was determined according to Dionisio-Sese et al. [60]. Relative water content (RWC) was measured following Gaxiola et al. [61]. Chlorophyll was extracted and measured as previously described by Lichtenthaler et al. [62]. Malondialdehyde (MDA) content was determined following the method of Heath et al. [63]. Root vitality was quantified with the triphenyltetrazolium chloride (TTC) method as previously described by Huo et al. [64]. Root hydraulic conductivity was determined using a pressure chamber (Model 1505D, PMS Instrument Company, Albany, OR, USA) as described previously [64].

### 4.4. Analysis of ROS Accumulation and Antioxidant Enzyme Activity

On day 6 of drought treatment, mature apple leaves were excised and stained with fresh solutions of 3,3′-diaminobenzidine (DAB) and nitro blue tetrazolium (NBT) to examine the accumulation of hydrogen peroxide (H_2_O_2_) and oxygen free radicals (O_2_^−^), respectively. The concentrations of H_2_O_2_ and O_2_^−^ and the activities of SOD (superoxide dismutase) and POD (peroxidase) were measured using test kits from Suzhou Comin Biotechnology Co., Ltd. (Suzhou, China).

### 4.5. Measurement of Photosynthetic Characteristics and Chlorophyll Fluorescence

The measurement of photosynthetic characteristics was performed on sunny days (09:00 to 11:00 h). Net photosynthetic rate (Pn) were determined using a portable Li-6400 system (Li-Cor, Inc., Lincoln, NE, USA) [42]. Chlorophyll fluorescence transients were measured using Open FluorCam FC 800-O (PSI, Brno, Czech Republic) and the quantum yield of PSII was measured using a Dual-PAM-100 system (Heinz Walz, Effeltrich, Germany) [65].

### 4.6. Determination of Insoluble and Oxidized Proteins

After 6 d of drought treatment, mature apple leaves were collected to examine the amount of insoluble and oxidized proteins. The concentrations of soluble, insoluble, and total proteins were measured with a protein assay kit (BCA), using bovine serum albumin as a standard. Oxidized proteins were measured through derivatization with 2,4-dinitrophenylhydrazine (DNPH), as previously reported by Shin et al. [21].

### 4.7. Measurement of Amino Acids and Flavonoid

Amino acids were measured according to earlier methods [30]. In brief, 0.1 g of leaf sample was extracted with 1 mL of 50% ethanol. After centrifugation at 12,000× *g* for 10 min, the filtered supernatants were diluted 20 times using methanol. The diluted solutions were used to determine the metabolites by high-performance liquid chromatography-mass spectrometry (HPLC-MS) (QTRAP5500; AB SCIEX, Washington, DC, USA). The measurement of flavonoid was conducted as reported previously by Zhou et al. [35].

### 4.8. Detection of Autophagic Structures

Mature apple leaves were harvested and immediately cut into small pieces (3 mm × 3 mm), then fixed with 0.1 M phosphate-buffered saline (PBS; pH 6.8) containing 2.5% glutaraldehyde for 12 h in the dark. Observations of the autophagic bodies were performed following the method of Sun et al. [38].

### 4.9. Statistical Analysis

Statistical data analysis was performed using SPSS 22.0 software (IBM, Chicago, IL, USA). The difference among means was determined through one-way ANOVA and Tukey’s multiple range test (*p* < 0.05).

## 5. Conclusions

In conclusion, *MdATG8i*OE apple plants exhibited enhanced tolerance to drought stress. The influence of *MdATG8i* on drought tolerance was associated with its role in promoting antioxidant defense, damaged protein degradation, flavonoid synthesis, and root water uptake. These changes in *MdATG8i*OE plants may be explained by increased autophagy under drought stress, as confirmed by the upregulation of other important *MdATGs* and greater accumulation of autophagosomes. The transgenic lines provide promising material for future breeding of apple varieties that are tolerant to drought stress.

## Data Availability

The data presented in this study are available in the article or Supplementary Material.

## Supplementary Materials

The following are available online at https://www.mdpi.com/article/10.3390/ijms22115517/s1.
