# Supplementary material for: Overexpression of MdATG8i Enhances Drought Tolerance by Alleviating Oxidative Damage and Promoting Water Uptake in Transgenic Apple
Source: Int J Mol Sci. 2021 May 24;22(11):5517. doi: 10.3390/ijms22115517 (PMC8197189; doi:10.3390/ijms22115517)
Supplement: Supplementary file 1 [file ijms-22-05517-s001.zip › Supplementary material.pdf]

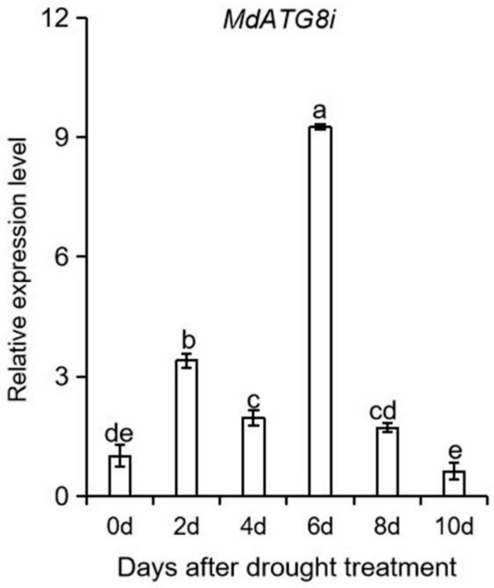

Figure S1. The relative expression of *MdATG8i* in the GL-3 apple plants exposed to drought stress. The data are the means of three replicates with SD.

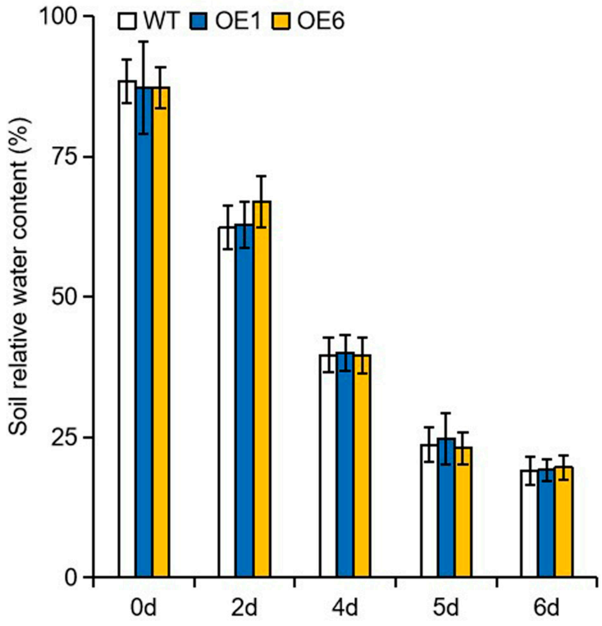

Figure S2. The soil relative water content of WT and *MdATG8i*OE plants following drought treatment. The data are the means of three replicates with SD.
