# Supplementary material for: Overexpression of MdATG8i Enhances Drought Tolerance by Alleviating Oxidative Damage and Promoting Water Uptake in Transgenic Apple
Source: Int J Mol Sci. 2021 May 24;22(11):5517. doi: 10.3390/ijms22115517 (PMC8197189; doi:10.3390/ijms22115517)
Supplement: Supplementary file 1 [file ijms-22-05517-s001.zip › Supplementary table.pdf]

Table. S1. Primers used in this study.

**Name**	**Sequence (5'-3')**	**Purpose**
MDH	F: CGTGATTGGGTACTTGGAAC<br>R:TGGCAAGTGACTGGGAATGA	Reference gene used in real-time PCR
q*ATG8i*	F: GCAGCAGGCTTCACTTGACTCC<br>R: GGAATCCATGCGACTGGCTGTT	Quantitative expression of *MdATG8i*
q*ATG3a*	F: AAGGGGGCGGAGATGGTTC<br>R: GCACTTAGAGACGAGGTTATCGC	Quantitative expression of *MdATG3a*
qATG3b	F: AGGGAGATGGTTTTGAAACAGA<br>R: ACTTAGAGACGAGGTTATCGC	Quantitative expression of *MdATG3b*
qATG4a	F: GCCTCCAAGCTGGCAGATGAATC<br>R: CCACTATCACCCAACGCATCACTG	Quantitative expression of *MdATG4a*
q*ATG7a*	F: GCGGATATGAGCAACCTTGGC<br>R: ATCAATAGGCGCAACGACATCA	Quantitative expression of *MdATG7a*
q*ATG7b*	F: ATCGGTAACAGGAGTAAGTCGG<br>R: TTTATCAAGCGCATGAAAGCCT	Quantitative expression of *MdATG7b*
q*ATG8c*	F: GCGTTCAAGATGGAGCACCCTC<br>R: CAGCCCTTTCCACAACCACTGG	Quantitative expression of *MdATG8c*
q*ATG8f*	F: TCGTAGACAATGTCCTCCCAGC<br>R: CCAAATGTGTTCTCGCCACTGT	Quantitative expression of *MdATG8f*
q*ATG9*	F: ACTTCATGCGTCAGCCTTCAGA<br>R: CGTTCCTCCAATCCAACCGTTG	Quantitative expression of *MdATG9*
q*ATG10*	F: TGGAACCAGCGAGTGGATGAAG<br>R: ACAACTGAGAGCCAAGACACCA	Quantitative expression of *MdATG10*
q*ATG11*	F: GAAGCGTTATTCACAACAACATCG<br>R: TTCCTCAAGTTCTCTTCCTTCACAA	Quantitative expression of *MdATG11*
qATG12	F: ACAGTGCATTCTCGCCAAACCC<br>R: CCCCATGCCATGGAGCAAGC	Quantitative expression of *MdATG12*
q*ATG18a*	F: ATGATTCCAGGCTTGCCTGCTTTG<br>R: TGCAGCAAAGTTCCGTCGAGAGTA	Quantitative expression of *MdATG18a*
qMdSOD	F: TGAAGGGTGTTGCTGTTCTCG<br>R: ATGAAGTCCAGGCTTGAGGC	Quantitative expression of *MdSOD*
qMdPOD	F: CCAACAAATGTGTCCCAAAAATG<br>R: CCTGGTCCGAGGTAAATAATCC	Quantitative expression of *MdPOD*
qMdPAL	F: GACCAAACGGTCAGACCCTCAATG<br>R: AGCCAAGCCAGAACCAACAGCAG	Quantitative expression of *MdPAL*
qMdCHS	F: TGGACGAAGTGAGGAGGAAGTCTG<br>R: CCACACTGTGAAGCACAACGGTCT	Quantitative expression of *MdCHS*
qMdCHI	F: CGGAGAATTGCGTTGTCTTTTGG<br>R: GTGATCCTTTGGGAGATTGTGTGA	Quantitative expression of *MdCHI*

MdPIP1;2	F: TTTCAACTTTCAAGACTCAA	Quantitative expression of *MdPIP1;2*
	R: GAACACTCACCATCAGAT	
MdPIP1;3	F: TGTACGTAATTAAGGGATTAAGCTG	Quantitative expression of *MdPIP1;3*
	R: ATCAGACGGACGGGATTG	
MdPIP1;4	F: GTGTGTTGTGGATGCGTTTC	Quantitative expression of *MdPIP1;4*
	R: TGATTCAGATGGATGGGATTGG	
MdPIP2;1	F: CCTTCTACCACCAATACATTC	Quantitative expression of *MdPIP2;1*
	R: TGATTATCTACAATTCCATAGCC	
